# SHM-Based Probabilistic Fatigue Life Prediction for Bridges Based on FE Model Updating

**DOI:** 10.3390/s16030317

**Published:** 2016-03-02

**Authors:** Young-Joo Lee, Soojin Cho

**Affiliations:** 1 School of Urban and Environmental Engineering, Ulsan National Institute of Science and Technology (UNIST), Ulsan 44919, Korea; ylee@unist.ac.kr; 2 Department of Civil Engineering, University of Seoul, Seoul 02504, Korea

**Keywords:** probabilistic fatigue life, fatigue life prediction, bridge fatigue, structural health monitoring, finite element model updating

## Abstract

Fatigue life prediction for a bridge should be based on the current condition of the bridge, and various sources of uncertainty, such as material properties, anticipated vehicle loads and environmental conditions, make the prediction very challenging. This paper presents a new approach for probabilistic fatigue life prediction for bridges using finite element (FE) model updating based on structural health monitoring (SHM) data. Recently, various types of SHM systems have been used to monitor and evaluate the long-term structural performance of bridges. For example, SHM data can be used to estimate the degradation of an in-service bridge, which makes it possible to update the initial FE model. The proposed method consists of three steps: (1) identifying the modal properties of a bridge, such as mode shapes and natural frequencies, based on the ambient vibration under passing vehicles; (2) updating the structural parameters of an initial FE model using the identified modal properties; and (3) predicting the probabilistic fatigue life using the updated FE model. The proposed method is demonstrated by application to a numerical model of a bridge, and the impact of FE model updating on the bridge fatigue life is discussed.

## 1. Introduction

Fatigue is one of the major causes of structural failure. In fact, many civil structures are exposed to repeated loading over their life cycles, and fatigue may lead to the failure of various types of structures, including bridges. A bridge is designed to survive for a certain period of time after it is constructed, but its status changes over its service life. It is thus essential to accurately predict the fatigue life in order to make decisions about effective bridge maintenance and retrofitting. However, this is a very challenging task, because fatigue life prediction for a bridge should be based on its current structural condition, which includes various sources of uncertainty, including material properties, anticipated vehicle loads and environmental conditions.

Probabilistic methods of fatigue life prediction have been studied by many researchers for various types of structures, such as offshore platforms and aircraft [1,2,3], and it has gained a lot of attention from researchers concerning bridges, as well. For example, Imam *et al.* [4] presented a probabilistic method for fatigue assessment of railway bridges and applied it to a typical short-span riveted railway bridge in England under train loading. Park *et al.* [5], Zhao *et al.* [6] and Madsen [7] estimated the fatigue reliability of bridges using a fracture mechanics approach. Lukić and Cremona [8] presented a probabilistic method for fatigue assessment using a crack growth model and applied the method to a typical steel bridge. However, these studies were based on the initial designs of bridges and the corresponding finite element models, which may not properly represent the current bridge condition. For accurate fatigue life prediction, the degradation of material properties in time, such as changes in the mass, stiffness and damping ratio, should be quantitatively considered, and the finite element (FE) model has to be updated accordingly.

Structural health monitoring (SHM) systems have often been used to overcome this issue and to evaluate the fatigue life of a bridge probabilistically based on the measurement of its current condition. Recently, various types of SHM systems are in use to monitor the long-term structural performance of bridges. Inspired by this, many researchers have proposed probabilistic approaches to estimate bridge fatigue life using the monitoring data. For example, Kwon and Frangopol [9] carried out fatigue reliability assessment of steel bridges using the probability density function of the equivalent stress range obtained from monitoring data. Ni *et al.* [10] developed a fatigue reliability model, using long-term monitoring data by integrating the probability distribution of the hot spot stress range with a continuous probabilistic formulation of Miner’s damage cumulative rule for fatigue life and reliability evaluation of steel bridges. Zhao and Haldar [11] proposed a fracture-based reliability model by considering the uncertainties in various measurements for nondestructive inspections, such as the initial crack size, the crack aspect ratio, the material properties and the number of stress cycles. In the abovementioned studies, however, measurement data by the monitoring system have typically been used to update the statistical information of random variables or the analytical model of fatigue crack growth. The data have rarely been applied to change the FE model, which helps with considering the current bridge condition.

This paper presents a novel approach to probabilistic fatigue life prediction for bridges using FE model updating based on SHM data. It has been reported that SHM data can be useful in evaluating structural performance and that structural changes that have occurred up to that time can be captured rationally using structural identification methods based on SHM data [12]. For example, SHM data can be used to estimate the degradation of an in-service bridge by updating an initial FE model of the bridge [13,14]. The proposed method involves three steps: (1) identifying the modal properties for a bridge, such as mode shapes and natural frequencies, using ambient vibration measurement; (2) updating the structural parameters of an initial FE model using the identified modal properties; and (3) predicting the fatigue life probabilistically using the updated FE model. The proposed method is demonstrated using a numerical model of a bridge described in Yi *et al.* [13] for the purpose of SHM-based FE model updating, and the impact of a change in the structural condition on the probabilistic fatigue life is discussed.

## 2. Probabilistic Fatigue Life Prediction Using FE Model Updating Based on SHM Data

### 2.1. Limit-State Function Formulations for Fatigue Failure

To calculate the probability of fatigue failure, it is necessary to construct the so-called limit-state function representing the failure event of interest as an analytical function of random variables and deterministic parameters. Many methods of fatigue reliability analysis for bridges have been developed starting from two deterministic fatigue models. The first model is Miner’s rule or the stress-life (S-N) curve [15], which is obtained by cycling loading of test specimens at a constant stress amplitude until visible cracking occurs. The second model is the Paris equation [16], which is based on fracture mechanics and is usually used to predict the propagation speed of an initial crack or defect. Lee and Song [17] derived a series of formulations for crack failure to estimate the structural risk of fatigue-induced sequential failure at the system level based on the Paris equation. In this study, the limit-state functions in Lee and Song [17] were modified for the purpose of evaluating the fatigue life of a bridge.

First, consider the following Paris equation [16], which is a widely-used crack-growth model:

(1) $$\frac{da}{dN}=C{\left(\Delta K\right)}^{m}$$

where *a* denotes the crack length, *N* denotes the number of loading cycles, *C* and *m* represent material properties and ∆*K* is the range of the stress intensity factor. The stress intensity factor range can be estimated by using Newman’s approximation [18] as follows:

(2) $$\Delta K=\Delta S\cdot Y(a)\cdot \sqrt{\pi a}$$

where Δ*S* denotes the range of the stress and *Y*(*a*) is the “geometry” function. Substituting Equation (2) into Equation (1), one can obtain the following:

(3) $$\frac{1}{{\left[Y(a)\sqrt{\pi a}\right]}^{m}}da=C\cdot \Delta S^{m}dN$$

The integration of Equation (3) from the initial condition to the current time point provides the relationship between the current crack length and the time duration as follows:

(4) $$\int_{a^{0}}^{a}\frac{1}{{\left[Y(a)\sqrt{\pi a}\right]}^{m}}da=C\cdot N\cdot \Delta S^{m}=C\cdot \nu_{0}\cdot T\cdot \Delta S^{m}$$

where *a*^0^ is the initial crack length, *N* is the total number of loading applications at frequency ν_0_ and *T* is the time duration. If it is assumed that a crack failure occurs when the crack length exceeds the critical crack length *a^c^*, the time required for crack growth from *a*^0^ to *a^c^*, *T*_0_, can be expressed as follows:

(5) $$T_{0}=\frac{1}{C\nu_{0}{\left(\Delta S_{0}\right)}^{m}}\int_{a^{0}}^{a^{c}}\frac{1}{{\left[Y(a)\sqrt{\pi a}\right]}^{m}}da$$

where *a*^0^ and Δ*S*_0_ are the initial crack length and stress range, respectively. The limit-state function for the failure of a member within a given time interval [0, *T_s_*] can be expressed as follows:

(6) $$g(X)=T_{0}-T_{s}=\frac{1}{C\nu_{0}{\left(\Delta S_{0}\right)}^{m}}\int_{a^{0}}^{a^{c}}\frac{1}{{\left[Y(a)\sqrt{\pi a}\right]}^{m}}da-T_{s}$$

where **X** denotes the vector of random variables. In structural reliability, *g*(**X**) ≤ 0 typically indicates the occurrence of a failure event.

After the initial FE model is updated based on SHM data, however, the stresses are changed, and the time required for fatigue failure also needs to be re-estimated accordingly. If the FE model updating is done at *T*^1^*_up_*, a recursive formulation of the time duration from that moment to the crack failure can be developed as follows. Consider an auxiliary “damage” function:

(7) $$\Psi (a)=\int_{a^{0}}^{a}\frac{1}{{\left[Y(a)\sqrt{\pi a}\right]}^{m}}da$$

From Equation (4), it is seen that:

(8) $$\Psi (a^{1})-\Psi (a^{0})=C\cdot \nu_{0}\cdot T_{up}^{1}\cdot {\left(\Delta S_{0}\right)}^{m}$$

(9) $$\Psi (a^{c})-\Psi (a^{1})=C\cdot \nu_{0}\cdot T_{1}\cdot {\left(\Delta S_{1}\right)}^{m}$$

where *a*^1^ and Δ*S*_1_ denote the crack length and stress, respectively, at the moment that the FE model is updated. Equations (8) and (9) represent the crack growth before and after the FE model is updated, respectively. Summing Equations (8) and (9), one obtains the following:

(10) $$\Psi (a^{c})-\Psi (a^{0})=C\nu_{0}T_{1}{\left(\Delta S_{1}\right)}^{m}+C\nu_{0}T_{up}^{1}{\left(\Delta S_{0}\right)}^{m}$$

Solving Equation (10) for *T*_1_,

(11) $$\begin{array}{ccc} T_{1} & = & \frac{1}{C\nu_{0}{\left(\Delta S_{1}\right)}^{m}}\left[\Psi (a^{c})-\Psi (a^{0})\right]-{\left(\frac{\Delta S_{0}}{\Delta S_{1}}\right)}^{m}T_{up}^{1}\\ & = & \frac{1}{C\nu_{0}{\left(\Delta S_{1}\right)}^{m}}\int_{a^{0}}^{a^{c}}\frac{da}{{\left[Y(a)\sqrt{\pi a}\right]}^{m}}-{\left(\frac{\Delta S_{0}}{\Delta S_{1}}\right)}^{m}T_{up}^{1} \end{array}$$

It is noteworthy that the ratio of the stresses Δ*S*_0_/Δ*S*_1_ incorporates the effect of the stress change obtained by the FE model updating. Similarly, if another FE model updating occurs at *T*^2^*_up_*, the time required for the crack failure after the second updating is derived as follows:

(12) $$T_{2}=\frac{1}{C\nu_{0}{\left(\Delta S_{2}\right)}^{m}}\int_{a^{0}}^{a^{c}}\frac{da}{{\left[Y(a)\sqrt{\pi a}\right]}^{m}}-{\left(\frac{\Delta S_{0}}{\Delta S_{2}}\right)}^{m}T_{up}^{1}-{\left(\frac{\Delta S_{1}}{\Delta S_{2}}\right)}^{m}T_{up}^{2}$$

After a mathematical induction, the following recursive formulation for multiple times of FE model updating can be derived:

(13) $$T_{j}=\frac{1}{C\nu_{0}{\left(\Delta S_{j}\right)}^{m}}\int_{a^{0}}^{a^{c}}\frac{da}{{\left[Y(a)\sqrt{\pi a}\right]}^{m}}-\sum_{i=1}^{j-1}{\left(\frac{\Delta S_{i}}{\Delta S_{j}}\right)}^{m}T_{up}^{i}$$

where *j* and *T_j_* denote the number of FE model updates and the time duration from the last SHM to the crack failure, respectively. The fatigue failure within a given time interval [0, *T_s_*] is then described as follows:

(14) $$g(X)=T_{up}^{1}+T_{up}^{2}+\cdot \cdot \cdot +T_{up}^{j}+T_{j}-T_{s}$$

Using Equation (14), the fatigue life of a bridge can be evaluated through repeated SHM and FE model updating. For the sake of simplicity, however, it is assumed in this research that the structural health monitoring is only performed once (*i.e.*, *j* = 1).

### 2.2. Component and System Reliability Analysis

A reliability analysis should be performed to calculate the probabilities of fatigue failure events using Equation (14). Numerous reliability analysis methods have been developed [19], and they can be grouped into two categories, sampling-based methods and analytical (or non-sampling-based) methods. The representative sampling-based method is Monte Carlo simulation (MCS), and the analytical methods are often represented by the first-order reliability method (FORM) and the second-order reliability method (SORM). A comprehensive review on these methods can be found in Melchers [19] and Der Kiureghian [20]. MCS is an easy method to use because it is conceptually straightforward. MCS needs to generate sample sets of random variables, and each set is used to run structural analysis and to check if the analysis result means failure or not. However, reliability analysis for fatigue life prediction generally requires repeated structural analyses, and it would be a daunting task if the analysis is conducted employing MCS, because MCS may require performing structural analyses for a large number of samples sets. In this research, FORM and SORM are used for component reliability analysis to overcome the disadvantages of using MCS for fatigue life prediction.

As mentioned previously, in a structural reliability problem, the limit-state function is termed *g*(**x**), and the event of interest (often called the “failure”) is expressed by *g*(**x**) ≤ 0, where **x** is a column vector of *n* random variables (*i.e.*, **x** = [*x*_1_, *x*_2_, …, *x_n_*]^T^) representing the uncertainties in the given problem. The probability of the event *P_f_* is expressed as follows:

(15) $$P_{f}=P\left[g\left(x\right)\leq 0\right]=\int_{g\left(x\right)\leq 0}f_{x}\left(x\right)dx$$

where *f***_x_**(**x**) is the joint probability density function (PDF) of **x**. By transforming the space of the random variables into the standard normal space, the probability *P_f_* can be expressed as follows:

(16) $$P_{f}=\int_{g\left(x\right)\leq 0}f_{x}\left(x\right)dx\int_{G\left(u\right)\leq 0}\phi_{n}\left(u\right)du$$

where *G*(**u**) = *g*(**T**^−1^(**u**)) is the transformed limit-state function in the standard normal space, φ*_n_*(·) denotes the *n-*th order standard normal PDF, **u** is the column vector of *n* standard normal variables and **T** is the one-to-one mapping transformation matrix that satisfies **u** = **T**(**x**).

**Figure 1:**
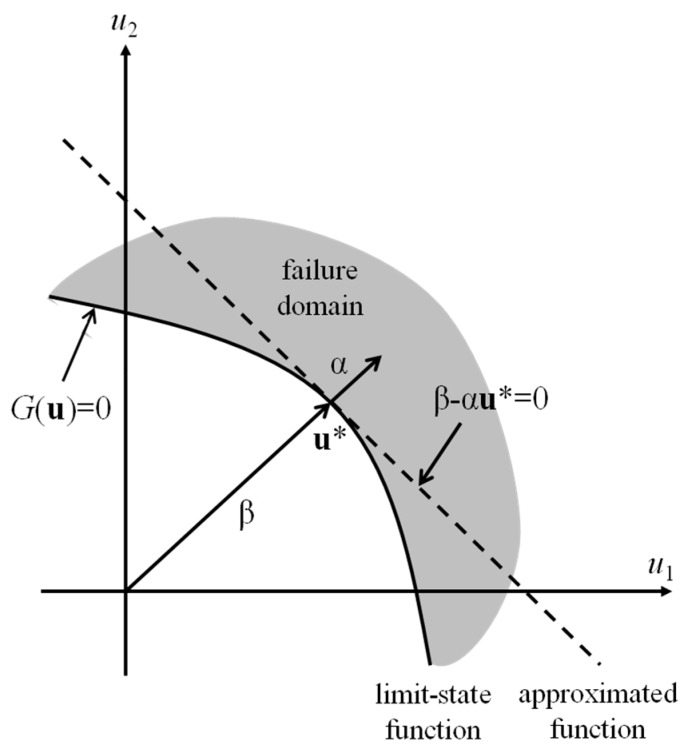
Linear approximation in the first-order reliability method (FORM).

In FORM, the probability (*i.e*., *P_f_* in Equation (15)) can be approximately calculated by use of the linearized function of *G*(**u**) at point u*, which is defined by the following constrained optimization problem:

(17) u*=arg min{∥u∥|G(u)=0}

where “arg min” denotes the argument of the minimum of a function and ∥·∥ is the L^2^ norm. As an example of the first-order approximation concept of the FORM, Figure 1 shows the approximated limit-state function in a two-dimensional space.

In the standard normal space shown in the figure, because the equal probability density contours are concentric circles centered at the origin, u* has the highest probability among all of the nodes in the failure domain *G*(**u**) ≤ 0. In this sense, u* is an optimal point and is commonly called the design point or most probable point (MPP).

The limit-state function approximated at MPP is written as follows:

(18) G(u)≅∇G(u*)(u−u*)=∥∇G(u*)∥(β−αu)

where ∇*G*(**u**) = [∂*G*/∂*u*_1_, …, ∂*G*/∂*u_n_*] denotes the gradient vector, **α** = −∇*G*(u*)/||∇*G*(u*)|| is the normalized negative gradient vector at MPP and β = −αu* is the reliability index. After FORM analysis, in this study, SORM is employed to achieve more accurate results than those from FORM. Der Kiureghian [20] provided more details about FORM and SORM.

For a complex structural system, a failure may be described as a system event that requires a system reliability analysis (SRA) [21,22]. In structural reliability, system events can be categorized into three groups, series system, parallel system and general system, and the detailed information can be found in Song and Kang [22]. However, it is noticeable that the component event probabilities and their correlations obtained from FORM are used for the probability computation of a system event [20,23]. Various SRA algorithms have been developed to compute the probability of this logical function of component events from the results of individual component reliability analyses [22]. The bridge example in this study consists of five girders, and the fatigue failure of each girder is defined by one component event, which means that there are five component failure events. In addition to their calculation, it is assumed that the bridge system fails if any of the five girders fail. The system failure event is described as a series event and requires a system reliability analysis. In this study, the multivariate normal integral method proposed by Genz [24] was employed to calculate the system probability. This method specializes in series and parallel system probability calculations and has been successfully tested on various structural and non-structural reliability problems [17,25].

### 2.3. Finite Element Model Updating Based on Structural Health Monitoring Data

**Figure 2:**
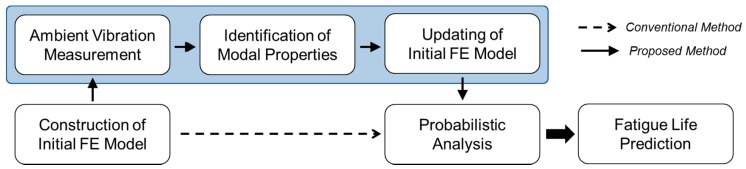
Flow chart of conventional and proposed fatigue life prediction procedures.

It is becoming increasingly important to monitor and evaluate the long-term structural performance of bridges and other structures, as well as their structural integrity, including the extent of material degradation and cracking. Many types of SHM systems have been instrumented and operated for this purpose for various types of structures. Yi *et al.* [13,14] recently presented a useful application of an instrumented SHM system for reliable seismic performance evaluation based on measured vibration data collected under ambient wind and traffic loadings. Since the structural degradation, including material deterioration and fatigue cracking, changes the structural stiffness, which substantially results in the changes of modal parameters estimated from SHM data, the fatigue life prediction followed by the FE model updating will become more accurate. The procedure consists of: (1) constructing an initial FE model of a target bridge based on its design drawings; (2) measuring the ambient vibration of the bridge under normal vehicle traffic; (3) identifying modal properties, including natural frequencies, mode shapes and modal damping ratios, from the measured acceleration data using an output-only modal identification method; (4) updating the linear structural parameters of the initial FE model using the modal properties identified; and, finally, (5) performing the probabilistic analysis of interest using the updated FE model. Figure 2 shows a schematic diagram of the proposed seismic performance evaluation procedure.

A downhill-simplex method [26] was employed in this study to find the optimal values of the structural parameters for an initial FE model based on the measured modal properties of a bridge. The objective function *J* in the optimization procedure represents the differences between the measured and calculated natural frequencies, and the constraint equations are constructed based on the differences between the measured and calculated mode shapes.

(19) $$J={\sum_{i=1}^{N_{m}}\left\{w_{i}\left(\frac{f_{i}^{c}-f_{i}^{m}}{f_{i}^{m}}\right)\right\}}^{2}\text{ subjected to }\left|\phi_{ji}^{c}-\phi_{ji}^{m}\right|\leq \varepsilon$$

where *f_i_* is the *i*-th natural frequency, *φ_ji_* denotes the *j*-th component of the *i*-th normalized mode shape *ϕ_i_*, *w_i_* and ε are the weighting factor for the *i*-th mode and the admissible error bound for the mode shape, respectively, and the superscripts “*m*” and “*c*” indicate data from the measurement and calculation of the FE model under updating, respectively. The details of the FE model updating procedure can be found in the following section and in Yi *et al.* [13,14].

## 3. Numerical Example

### 3.1. Example Bridge: Samseung Bridge

**Figure 3:**
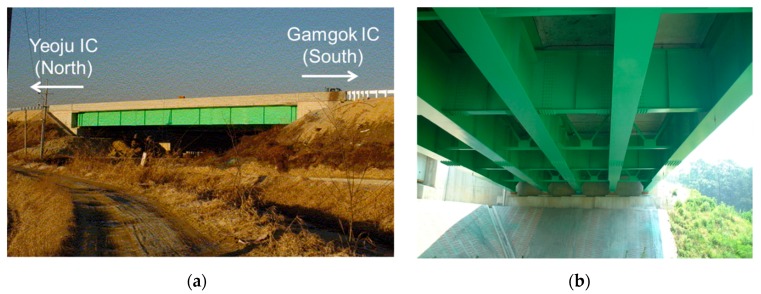
(**a**) Front view and (**b**) bottom view of Samseung Bridge, Korea.

The proposed fatigue life estimation based on the FE model updating was validated for a real bridge on a highway of Korea, Samseung Bridge shown in Figure 3. The bridge was used as a testbed of the FE model updating in Yi *et al.* [13] with extensive field tests. The bridge is on the test road of Korea Expressway Corporation (KEC) that was built parallel to the main lane of the Jungbu Inland Expressway of Korea. The test road was constructed in 2002 between the Yeoju Interchange (IC) north and Gamgok IC south, and so was the bridge.

**Figure 4:**
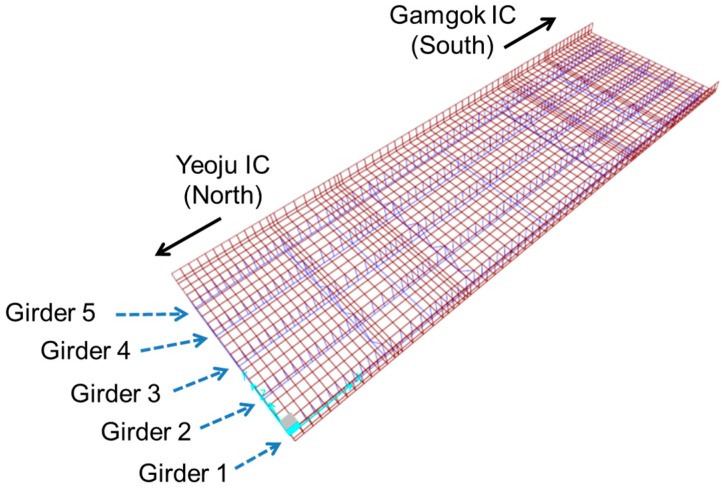
FE model of Samseung Bridge developed using SAP2000. IC, Interchange.

Samseung Bridge is a single-span steel-plate girder bridge with a span length of 38.8 m. It is composed of five main steel girders, floor beams and a concrete slab. Based on the design drawings of the bridge, an initial FE model was constructed using SAP2000, as shown in Figure 4. In the figure, the shell elements (in red) and frame elements (in blue) represent the concrete slab and steel girders, respectively. The five girders are labeled Girder 1 through Girder 5, numbered from the bottom to the top.

### 3.2. FE Model Updating

**Figure 5:**
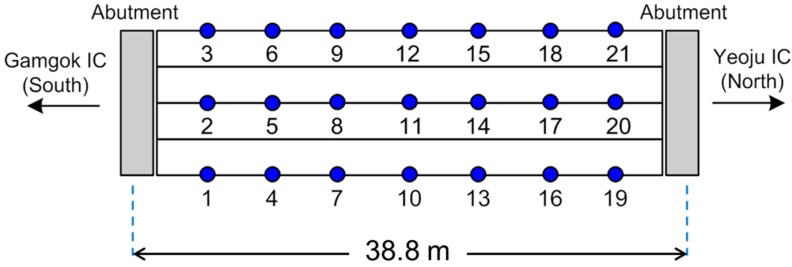
Location of accelerometers in ambient vibration tests.

The initial FE model of Samseung Bridge was updated in Yi *et al.* [13] based on the ambient vibration measurements. A series of ambient vibration tests were carried out on the bridge in four different seasons: (1) August 2004; (2) December 2004; (3) July 2005; and (4) February 2006. For the ambient vibration tests, 21 accelerometers were installed on the bridge, as shown in Figure 5. The wind and traffic on the adjacent bridge were the main vibration sources during the ambient vibration tests. Ambient vibrations were measured for 30 min at a sampling frequency of 200 Hz. A low-pass filter with a cut-off frequency of 90 Hz was utilized to avoid aliasing. To validate the result of the FE model updating, loading tests with varying truck weights and speeds were carried out after each ambient vibration test. The measured deflections by the loading trucks were compared to the simulated ones in the updated FE models to see the validity of the FE model updating. More details of the field test and FE model updating can be found in Yi *et al.* [13].

The initial FE model was updated using the extracted modal properties from the ambient vibration data. The modal properties, such as natural frequencies and mode shapes, were extracted from ambient vibration data using a well-known output-only modal identification method, the stochastic subspace identification method [27]. With the objective function in Equation (19), the downhill simplex method [26] was employed as an updating algorithm, and the analysis engine of SAP2000 was used to iteratively calculate the modal properties of the FE model under updating. To avoid possible ill-posedness during the updating, model updating was processed in two steps with different updating parameters, as tabulated in Table 1. The initial FE model was updated using nine updating parameters, and the model was further updated using 31 detailed updating parameters, as shown in Table 1. The updating parameters include rotational spring constants at the supports, Young’s modulus of the concrete slab, the second moments of inertia for the five main girders and the equivalent second moments of inertia and torsional coefficients for the nine floor beams.

After updating the FE model, the natural frequencies of the initial FE model and updated FE model were compared to the measured ones. The comparison shows that the natural frequencies of the FE model become close to the measured values after the updating. Furthermore, the simulated displacements on the updated FE model showed high agreement with the displacements measured from the loading tests. The comparisons are illustrated in Figures 15 and 16 of Yi *et al.* [13].

In this research, the initial and updated FE models of Samseung Bridge were used as a numerical example to evaluate its probabilistic fatigue life based on both the initial and updated FE models. Among various updated FE models, the updated model using a test data in July 2005 (named S5-2 in Yi *et al.* [13]) was selected based on its good updating result.

### 3.3. Statistical Parameters

**Figure 6:**
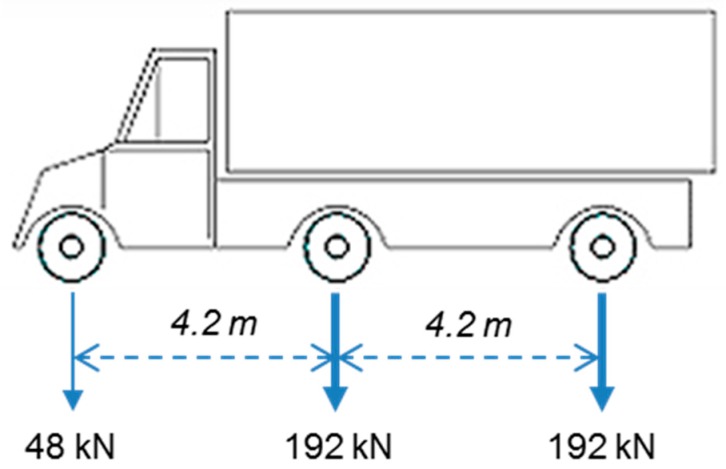
Vehicle load model (DB-24) of the Korea Highway Bridge Design Specification (KHBDS).

To evaluate the fatigue life of a bridge, a fatigue load model should be developed. In many existing studies, techniques, such as weigh-in-motion (WIM) measurements, direct sensor reading and rain flow counting based on actual passing vehicles, have been used to obtain the magnitudes and frequencies of fatigue loadings. However, this type of field test was not conducted on the Samseung Bridge. In addition, as previously mentioned, SHM data on the bridge were collected for 30 min only. Therefore, in this study, the fatigue load was modeled using the vehicle load model (DB-24) of the Korea Highway Bridge Design Specification (KHBDS) by the Ministry of Construction and Transportation in Korea [28], as shown in Figure 6.

Using the load model, an FE analysis was performed with the initial and updated FE models to determine the maximum stress ranges in the five girders. Because the stresses were obtained using static analyses, they were multiplied by the impact factor *I* given by Equation (20) to account for the dynamic effect of vehicle loads.

(20) $$I=\frac{15}{40+L}\leq 0.3$$

where *L* is the span length of the bridge in meters. In the FE models constructed using SAP2000, the span length was assumed to be 38.8 m. Thus, the impact factor is 1.19.

Table 2 lists the maximum stress ranges of the five girders obtained from the initial and updated FE models. As shown in the table, the stress values obtained from the updated model are smaller to those from the initial model. It is noteworthy that the stress values are symmetrical with the center of Girder 3 in the initial FE model, reflecting its symmetry, but slightly asymmetrical in the updated model. This is because the structural parameters change during the FE model updating process. The table also shows that the stress values from the updated FE model are relatively small compared to those from the initial model. This is because the model updating was carried out using the measurement in 2005 (only three years after the bridge construction), which means that the bridge is still expected to be in good condition.

### 3.4. Random Variables and Deterministic Parameters

Accurate results of fatigue life prediction would be expected if the statistical information of random variables could be obtained from test and observation on the example, which was not feasible in this paper. Instead, the statistical information is determined through a comprehensive literature survey [17,25,29,30,31,32,33]. The uncertainty of *C* in the Paris equation and that of the initial crack length *a*^0^ are considered to be random variables with mean values of 2.18 × 10^−13^ mm/cycle/(MPa·mm)*^m^* and 0.1 mm, respectively, and the coefficients of variation (COVs) are 0.2 and 0.1, respectively. The parameter *m* in the Paris equation can also be considered as a random variable. However, a preliminary analysis showed that the consideration gave a negligible impact to the result of life prediction for a large amount of additional time costs, so only *C* is considered as a random variable in this example. The uncertainties of the stresses are also introduced using a load scale factor *S*, whose mean and COV are assumed to be 1.0 and 0.1, respectively. It is assumed that the initial crack length *a*^0^ follows an exponential distribution, whereas the other parameters follow a lognormal distribution. The statistical properties of the random variables in this numerical example are summarized in Table 3.

All of the random variables are assumed to be statistically independent of each other, except in the following cases: (1) between the Paris equation parameter *C* values of the five girders (correlation coefficient: 0.6); and (2) between the initial crack lengths (*a*^0^) of the five girders (correlation coefficient: 0.6). The correlation coefficients in these cases are not known, so they were initially assumed to be 0.6, which indicates that Girders 1–5 were manufactured by the same manufacturer and that their material properties are thus highly correlated. In addition, a parametric study with various correlation coefficients was performed to investigate the effects of these correlations on the fatigue life.

In addition, the following deterministic parameters were used: the half flange width (W): 650 mm; the flange thickness (*f_th_*): 30 mm; the critical crack length (*a^c^*): 30 mm; and the time of the SHM test (*T*^1^*_up_*): four years. The average daily truck traffic (ADTT) was assumed to be 5388/day, based on actual passing truck data provided by the Korea Expressway Corporation. For the geometry function *Y*(*a*) in Equation (2), the following function from Wang *et al.* [34] for I-beams is introduced:

(21) $$Y=\frac{1.0-0.5\left(\frac{a}{W}\right)+0.37{\left(\frac{a}{W}\right)}^{2}-0.044{\left(\frac{a}{W}\right)}^{3}}{\sqrt{1-\frac{a}{W}}}$$

### 3.5. Analysis Results

**Figure 7:**
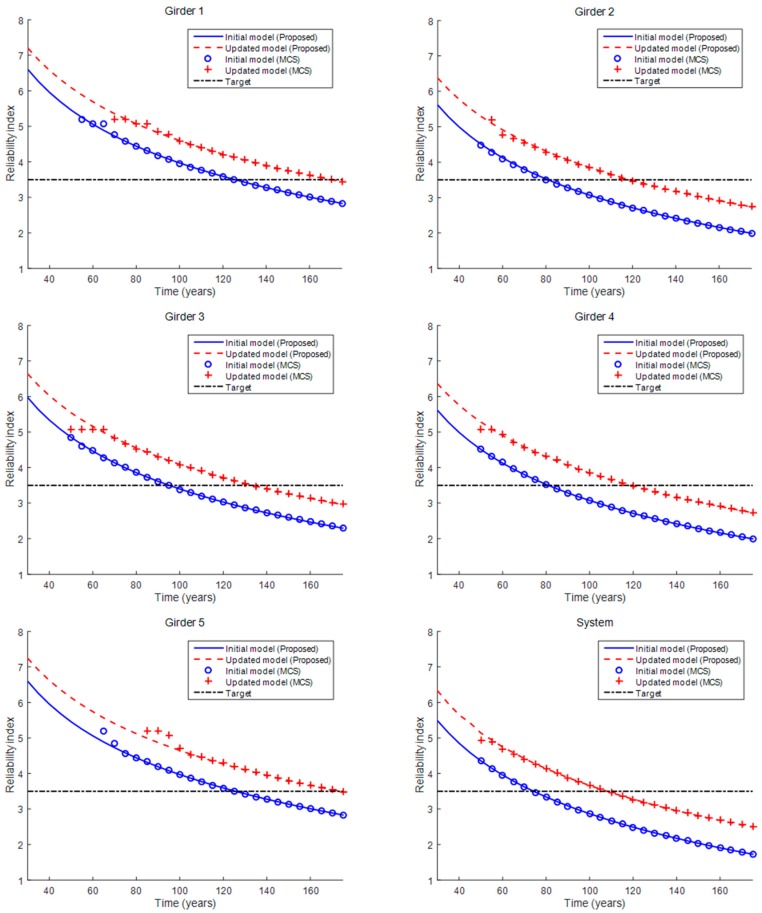
Reliability indices of girders and the bridge system using the proposed method and Monte Carlo simulation (MCS).

To compare the fatigue life assessment results from the initial and updated FE modes, Figure 7 was prepared to show the reliability indices for various service times for the five girders and the bridge system, as obtained from the proposed method and MCS. As previously mentioned, the system failure event was assumed to occur when any of the five girders failed. Obviously, the reliability indices of the bridge decrease with increasing service life, which means that the probability of failure increases with increasing use of the bridge. In addition, the reliability index of the bridge system is smaller than those of the individual girders because of the event definition.

Figure 7 also shows that the reliability indices from the updated FE model are larger than those from the initial model at both the component and system levels. This is because the stress values are relatively small with the updated FE model, as shown in Table 2. The American Association of State Highway and Transportation Officials (AASHTO) recommends, in the AASHTO Bridge Design Code, a target reliability index of 3.5 (*i.e.*, a failure probability of 2.33 × 10^−4^) with a service life of 75 years for steel and prestressed concrete components. The fatigue lives of Girders 1–5 and the bridge system were estimated using the target reliability index (*i.e*., the black lines in Figure 6), as listed in Table 4. With the updated FE model, the fatigue lives of the girders and bridge system were estimated to be much greater, and all of them meet the AASHTO requirement, with fatigue lives longer than 75 years.

For verification purposes, MCS was performed with 10^7^ samples, and the results obtained with the proposed method match those obtained from the MCS, except in the range of relatively large reliability index values, where accurate results cannot be expected, even with MCS using 10^7^ samples in nature.

Lastly, in the results summarized above, the correlation coefficient ρ between the values of the Paris equation parameter *C* and between the initial crack lengths *a*^0^ of the five girders were assumed to be 0.6, which accounts for the high dependency due to the same manufacturer assumption. To investigate the effect of the correlation coefficient, the fatigue life of the bridge system was evaluated using a range of correlation coefficient values, as a parametric study. As Table 5 shows, the fatigue life increases by 2–4 years as the correlation coefficient increases, which means that the effect of the correlation coefficient is not very significant. This is because, although the failure event of the bridge system is assumed to occur if any of the five girders fails, the system failure event is actually dominated by the failure events of Girders 2 and 4, as shown in Figure 7 and Table 4.

## 4. Conclusions

In this paper, a new approach is proposed for SHM-based prediction of the fatigue life of a bridge using an FE model updating method. The proposed method consists of three steps: (1) identifying the modal properties of a bridge, such as the mode shapes and natural frequencies, based on the ambient vibration measurement; (2) updating the structural parameters of an initial FE model using the identified modal properties; and (3) predicting the fatigue life probabilistically using the updated FE model. After building an initial FE model of a bridge, the optimal values of the structural parameters, which minimize the difference of the natural frequencies between the measurement and the FE model, are identified using the downhill simplex method to obtain the updated FE model.

In addition, new limit-state formulations were derived to express the crack failure and predict the probabilistic fatigue life with updated stress values. These formulations allowed us to evaluate the fatigue life of a bridge with repeated SHM and FE model updating. To demonstrate the proposed method, it was applied to a numerical model of the Samseung Bridge, whose FE model updating was already addressed in a previous study. The reliability indices of the five girders and the bridge system determined from the updated FE model were larger than those determined from the initial FE model, because the stress levels were relatively low for the updated model. As a result, the fatigue lives of the girders and bridge system were estimated to be much longer, which indicates that the bridge is still in good condition. The reliability index results obtained from the proposed method were verified using MCS. Furthermore, as a parametric study, the fatigue life of the bridge system was evaluated using a range of correlation coefficients to investigate the effect of the correlation coefficient, and it was found that the effect of the correlation coefficient on the predicted bridge fatigue life was not very significant. In conclusion, the proposed method was shown to make it possible to predict the fatigue life of a bridge probabilistically, based on its current condition, using the SHM data and updating of the corresponding FE model.

## Figures and Tables

**Table 1:** Updating parameters of Samseung Bridge (modified from [13]).

Members	Updating Parameters	Count	
		First Step	Second Step
Support	Rotational Spring Constant	1	2
Concrete Slab	Young’s Modulus	1	1
Main Girder	Second Moment of Inertia	5	5
	Torsional Coefficient	0	5
Floor Beam	Second Moment of Inertia	1	9
	Torsional Coefficient	1	9
Total		9	31

**Table 2:** Maximum stress ranges of girders from initial and updated FE models.

Stress (MPa)	Girder 1	Girder 2	Girder 3	Girder 4	Girder 5
Initial FE model	18.24	20.77	20.03	20.77	18.24
Updated FE model	15.95	17.52	17.11	17.52	15.83

**Table 3:** Statistical properties of random variables.

Random Variables (RVs)	Mean	COV	Distribution Type	Number of RVs
Paris law parameter ©	2.18 × 10^−13^ (mm/cycle/(MPa·mm)*^m^*)	0.2	Lognormal	5
Initial crack length (*a*^0^)	0.1 (mm)	1.0	Exponential	5
Live load scale factor (*S*)	1	0.1	Lognormal	1

**Table 4:** Fatigue life estimated from initial and updated FE models.

Fatigue Life (Years)	Girder 1	Girder 2	Girder 3	Girder 4	Girder 5	System
Initial FE model	125.6	81.2	95	81.2	125.6	74.3
Updated FE model	170	119.4	133.6	118.3	175	108

**Table 5:** Fatigue life of the bridge system for various correlation coefficients.

Bridge System Fatigue Life (Years)	*ρ* = 0.0	*ρ* = 0.2	*ρ* = 0.4	*ρ* = 0.6	*ρ* = 0.8
Initial FE model	73	73.3	73.7	74.3	75.7
Updated FE model	106	106.5	107	108	110
